# Patient consent preferences on sharing personal health information during the COVID-19 pandemic: “the more informed we are, the more likely we are to help”

**DOI:** 10.1186/s12910-022-00790-z

**Published:** 2022-05-20

**Authors:** Sarah Tosoni, Indu Voruganti, Katherine Lajkosz, Shahbano Mustafa, Anne Phillips, S. Joseph Kim, Rebecca K. S. Wong, Donald Willison, Carl Virtanen, Ann Heesters, Fei-Fei Liu

**Affiliations:** 1 Radiation Medicine Program, Princess Margaret Cancer Centre, 700 University Avenue, Toronto, ON M5G 2M9 Canada; 2 Department of Radiation Oncology, University of Toronto, Toronto, ON Canada; 3 Department of Biostatistics, Princess Margaret Cancer Centre, Toronto, ON Canada; 4 Novo Nordisk, Plainsboro, USA; 5 Department of Medicine, University Health Network, Toronto, ON Canada; 6 Institute of Health Policy, Management and Evaluation, University of Toronto, Toronto, ON Canada; 7 University Health Network Digital, Toronto, ON Canada; 8 Department of Bioethics, University Health Network, Toronto, ON Canada; 9 Joint Centre for Bioethics, University of Toronto, Toronto, ON Canada

**Keywords:** Consent during pandemics, COVID-19, Patient consent preferences, Data sharing during pandemics, Research ethics

## Abstract

**Background:**

Rapid ethical access to personal health information (PHI) to support research is extremely important during pandemics, yet little is known regarding patient preferences for consent during such crises. This follow-up study sought to ascertain whether there were differences in consent preferences between pre-pandemic times compared to during Wave 1 of the COVID-19 global pandemic, and to better understand the reasons behind these preferences.

**Methods:**

A total of 183 patients in the pandemic cohort completed the survey via email, and responses were compared to the distinct pre-pandemic cohort (n = 222); all were patients of a large Canadian cancer center. The survey covered (a) broad versus study-specific consent; (b) opt-in versus opt-out contact approach; (c) levels of comfort sharing with different recipients; (d) perceptions of commercialization; and (e) options to track use of information and be notified of results. Four focus groups (n = 12) were subsequently conducted to elucidate reasons motivating dominant preferences.

**Results:**

Patients in the pandemic cohort were significantly more comfortable with sharing all information and biological samples (90% vs. 79%, *p* = 0.009), sharing information with the health care institution (97% vs. 83%, *p* < 0.001), sharing information with researchers at other hospitals (85% vs. 70%, *p* < 0.001), sharing PHI provincially (69% vs*.* 53%, *p* < 0.002), nationally (65% vs. 53%, *p* = 0.022) and internationally (48% vs. 39%, *p* = 0.024) compared to the pre-pandemic cohort. Discomfort with sharing information with commercial companies remained unchanged between the two cohorts (50% vs. 51% uncomfortable, *p* = 0.58). Significantly more pandemic cohort patients expressed a wish to track use of PHI (75% vs. 61%, *p* = 0.007), and to be notified of results (83% vs. 70%, *p* = 0.012). Thematic analysis uncovered that transparency was strongly desired on outside PHI use, particularly when commercialization was involved.

**Conclusions:**

In pandemic times, patients were more comfortable sharing information with all parties, except with commercial entities, where levels of discomfort (~ 50%) remained unchanged. Focus groups identified that the ability to track and receive results of studies using one’s PHI is an important way to reduce discomfort and increase trust. These findings meaningfully inform wider discussions on the use of personal health information for research during global crises.

**Supplementary Information:**

The online version contains supplementary material available at 10.1186/s12910-022-00790-z.

## Introduction

Rapid access to patient health information (PHI) by medical researchers during pandemics is of paramount importance to advance the clinical understanding of novel pathogens and enable the swift development of life-saving treatments and vaccines. Health-related organizations must be able to develop patient consent policies and processes that are sufficiently nimble to facilitate timely access while adhering to ethical standards defined by governing bodies (e.g. Canada’s Tri-Council Policy Statement: Ethical Conduct for Research Involving Humans, TCPS2, 2018 [1]; European Union’s General Data Protection Regulation, GDPR, 2018 [2]; United States’ National Institute of Health Data Sharing Policy, 2020 [3]), as well as institutional Research Ethics Boards (REBs). While REBs may be able to provide exemptions from requiring consent for some studies during pandemics and other exceptional circumstances (TCPS2, 2018; Article 3.7A) [1], investigating the consent needs and preferences of patients during a pandemic is pivotal to ensuring transparent patient-centered policies that maximize rapid research whilst meeting patient needs.

Pandemics pose unique challenges to traditional informed consent processes, which are ordinarily conducted by in-person, face-to-face conversations with clinicians or research coordinators, with direct opportunities to ask questions and sign physical consent forms. While pandemic policies and approaches will differ across international contexts, in our Canadian context, clinical activities were reduced and entry into hospitals was restricted to protect patients and health care workers. Unfortunately, this also impeded opportunities to engage in the dialogue necessary for valid informed consent. Strict pandemic visitor policies limiting who might be considered a patient’s essential care partner also had the potential to reduce a patient’s level of comfort with consenting to treatments or research studies. Solutions proposed and enacted to overcome these challenges included utilizing digital tools, documenting verbal consent, using electronic informed consent, and the employment of digital HIPAA-compliant tools such as e-mail surveys or telehealth assessments [4]. Leveraging digital platforms for telehealth and consent processes may have presented additional benefits to research by enabling the inclusion of a larger number of eligible patients who might otherwise be excluded due to geographic, mobility, or language barriers. However, there is little understanding of how these changes may affect the process of informed consent, and whether patients’ overall comfort with participation and technology-enabled consent options was increased or decreased under conditions where the vulnerability associated with the experience of critical illness was coupled with pandemic psychosocial stressors.

There is a paucity of literature on patient preferences for research participation during a pandemic. In fact, Gobat et al. (2015 [5]; 2017 [6]; 2019 [7]) have repeatedly highlighted that while patients are primary contributors and beneficiaries of pandemic-related clinical research, systematic studies regarding their views on research participation during a pandemic is lacking. Their preliminary explorations of mostly European patients indicated that while 74.8% thought “special rules” should apply to pandemic-relevant research, most (58.4%) still preferred standard enrollment procedures, such as prospective written informed consent, with only 38.6% opining that simplified procedures would be acceptable. It was also observed that the patients’ level of trust in both health professionals and the government was predictive of their willingness to participate in pandemic-relevant research [7]. Furthermore, while use of routinely collected data and clinical samples for pandemic-relevant research without explicit prior consent was supported in principle, it was found less acceptable when a profit motive was perceived [6]. Therefore, it is clear that further empirical research in this area is warranted.

In a prior study by our group [8], we sought to acquire insight to the contemporary and specific consent needs of cancer patients at a large Canadian academic hospital to inform institutional consent policies. In that study (conducted in radiation oncology clinics prior to the COVID-19 pandemic), the majority of the 222 participants (83%) were willing to share PHI with researchers at our own institution, though many preferred a more transparent and reciprocal consent process [8]. Prior to the COVID-19 pandemic, the majority of surveyed patients (63%) desired to be asked for permission before being entered into a research contact pool; 38% preferred study-specific consent (i.e., be given information on each study and decide each time vs. 56% preferring one-time broad consent for all studies; 6% would not share at all), and approximately half of patients were uncomfortable sharing PHI with commercial enterprises [8]. Most patients desired the option to track PHI usage (61%), with the highest proportion reported by the youngest group of patients (≤ 49 years: 71%); and the majority wished to be notified regarding study results (70%). The level of comfort in sharing PHI was highest for those within one’s own health organization (83%), followed by researchers at other academic institutions (70%), and followed by not-for-profit companies such as health charities (57%). By a substantial margin, the lowest level of comfort was reported for sharing with for‐profit commercial companies (27%). To build on both these findings and the aforementioned preliminary explorations of consent preferences during pandemics, the objectives of the present study were to: (a) ascertain the preferences of patients during the COVID-19 global pandemic to uncover potential differences between pre-pandemic and pandemic cohorts, and (b) acquire a deeper understanding of the reasons underlying these consent preferences.

## Methods

This study employed a mixed methods design with both quantitative (i.e. survey), and qualitative (i.e. focus group) strands, which were intricately linked in that the focus group guide was designed to further explore key findings from our pre-pandemic and pandemic cohort survey responses (e.g. who should decide if health information is shared, contact pool permissions, tracking future use, and sharing for commercial purposes).

### Surveys

A study information letter and link to a 10-min online survey (Additional file 1: Appendix 1) were digitally sent to patients in Canada’s Princess Margaret (PM) Cancer Centre’s Virtual Care database who had consented to email contact (~ 1000 patients in total) during Wave 1 of the COVID-19 pandemic. This survey design was based on existing evaluations of patient consent preferences across Canada and internationally (e.g. [9–13]), and was developed with the input of experts from medicine, bioethics, digital technology, and public health policy. This exact survey was administered in-person to a distinct cohort of 222 patients at the same Canadian cancer center during calendar year 2019 [8], allowing the unique opportunity to glean insights into how patient perceptions for sharing health information for general use outside clinical care might differ during ordinary times versus a global pandemic. Key survey items focused on: (a) broad versus study-specific consent; (b) opt-in versus opt-out approaches for a research contact pool; (c) levels of comfort in sharing with different types of recipients; (d) perceptions related to commercialization; and (e) options to track use of information and notification of study results.

Questionnaire responses, stratified by cohort, were summarized using descriptive statistics. Differences in response distribution between cohorts were analyzed using the Chi-square test. To evaluate whether differences in response distributions remained statistically significant after accounting for patient demographics, multivariable logistic and proportional odds ordinal regression models were fitted to the data. The ordinal regression models were used for questions evaluating comfort level (Q7 to Q17), with comfort level modelled as a three-level ordinal outcome: uncomfortable/very uncomfortable versus neutral versus comfortable/very comfortable. Therefore, an odds ratio > 1 denoted increased odds of reporting comfort. Logistic regression models were used for questions not assessing comfort levels (Q1 to Q5, Q18, Q19). The models adjusted for age group (< 49 vs. 50 to 74 vs. 75+), sex, treatment phase and education levels (completed post-secondary versus not completed post-secondary education). The proportional odds assumption was evaluated for the ordinal regression models.

### Focus groups

All participants who completed the virtual survey during the COVID-19 pandemic were invited by a clinical research coordinator email to participate in one virtual 60–90-min focus group. The focus group guide was pilot tested with a preliminary focus group of three patient partners to ensure that the topics were presented in a clear and balanced fashion. Four focus groups were subsequently conducted with 12 participants (response rate 6%) by the study’s lead author (ST; a Scientific Associate in the Radiation Medicine Program with no clinical relationship to the participants). For each focus group, participants were read an introductory script informing them of the study purpose and indicating that their participation constituted consent to participate in the study. The focus group guide (Additional file 2: Appendix 2) was built around four key questions: (1) Who should decide if PHI is shared?; (2) How should we contact patients to participate in research?; (3) What information do you need about future studies that propose to use your PHI?; and (4) How do you feel about commercial companies using your PHI?

Interviews were audio-recorded and transcribed verbatim, with all names and identifying features removed. Thematic analysis was employed to analyze the data; transcripts were read fully to capture holism, and the text was grouped into meaningful pieces of information known as meaning units (MUs), which were grouped based on similar features to create prominent themes and sub-themes [14]. Three members of the research team reviewed the transcripts and codes to ensure inter-rater reliability; and discussion ensued until consensus was reached to resolve any discrepancies. Constant comparison method [15] was used to ensure consistent classification of MUs. The ethnographic methodology of grounded theory [16] served as the framework for analysis as we aimed to construct theory from the data and allow themes to emerge iteratively, rather than being classified based on existing theories or pre-established categories. The Research Ethics Board (REB) of the University Health Network (UHN) provided full ethics approval of this study, and all methods were carried out in accordance with relevant guidelines and regulations. All study participants were provided with a detailed Study Information Letter to ensure informed consent, and were given the opportunity to ask any questions prior to consenting to participate.

## Results

### Surveys

#### Cohort characteristics

A total of 183 and 222 patients in the pandemic and pre-pandemic cohort respectively, completed the survey (Table 1). The pandemic cohort contained more patients at the treatment stage (57% vs. 39%, *p* < 0.001), had fewer patients in the oldest age group of 75+ years (13% vs. 22%, *p* < 0.001), and was more likely to have completed a college or university degree (76% vs. 63%, *p* < 0.001). The sexes were similarly distributed across the two cohorts, with 50% and 49% of the pandemic and pre-pandemic cohorts identifying as female.

**Table 1:** Baseline characteristics, stratified by cohort

Demographic	Pandemic (N = 183)	Pre-Pandemic (N = 222)	*p* value
Treatment stage			
Pre-treatment	10 (5%)	22 (10%)	**0.001**
Treatment	104 (57%)	87 (39%)	
Follow-up	69 (38%)	113 (51%)	
Age			
18–34	2 (1%)	10 (5%)	**0.005**
35–49	17 (9%)	28 (13%)	
50–74	139 (76%)	132 (59%)	
75+	24 (13%)	48 (22%)	
Rather not say	1 (1%)	4 (2%)	
Sex			
Male	90 (49%)	112 (51%)	0.94
Female	92 (50%)	108 (49%)	
Rather not say	1 (1%)	2 (1%)	
Education			
High school or less	15 (8%)	42 (19%)	**< 0.001**
Some post-secondary training	29 (16%)	35 (16%)	
Completed trade/college diploma	32 (17%)	36 (16%)	
Completed university degree	48 (26%)	62 (28%)	
Completed post-graduate degree	59 (32%)	41 (18%)	
Rather not say	0 (0%)	6 (3%)	

The bold values are statistically significant

#### Consent preferences by cohort

**Fig. 1:**
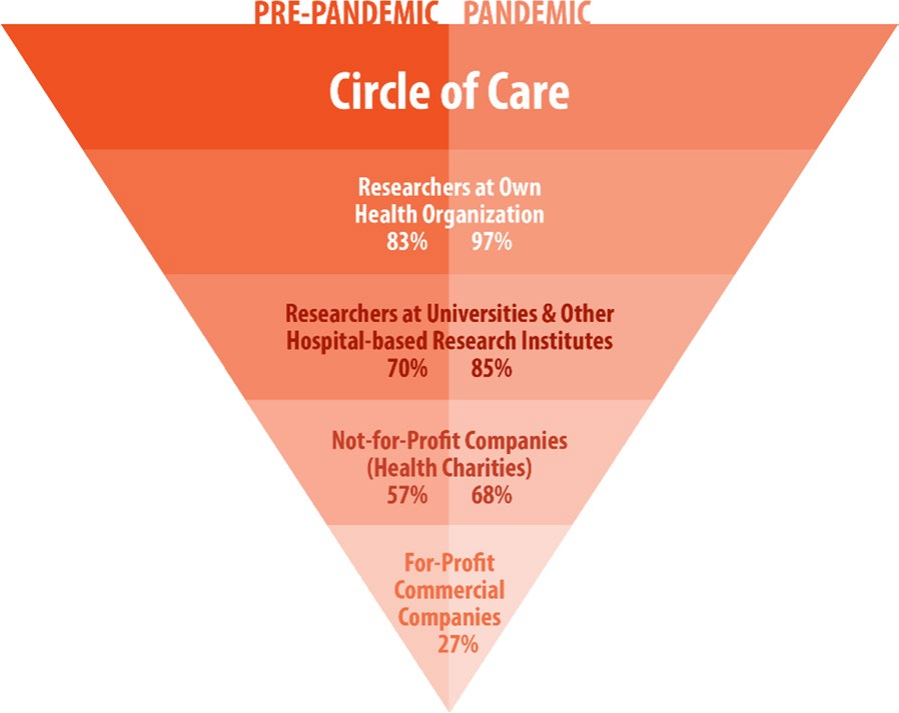
Percentage of patients reporting comfort sharing PHI as a function of recipient comparing pre-pandemic versus pandemic cohorts. Inverted pyramid chart showing the progressive decline in percentage of participants reporting comfort sharing with recipients outside the circle of care from within one’s own health organization to for-profit commercial companies, and that during a pandemic, participants felt more comfortable sharing with all recipients except for-profit commercial companies

More patients in the pandemic cohort were comfortable with sharing all health information and biological samples (Q2, 90% vs. 79%, *p* = 0.009). They were also more comfortable sharing information with their own health care institution (Q7, 97% vs. 83%, *p* < 0.001), and sharing information with researchers at other hospitals (Q8, 85% vs. 70%, *p* < 0.001). Patients in the pandemic cohort were also more comfortable sharing information provincially (Q12, 69% vs. 53%, *p* < 0.002), nationally (Q13, 65% vs. 53%, *p* = 0.022) and internationally (Q14, 48% vs. 39%, *p* = 0.024; Table 2). Comfort in sharing PHI with commercial companies remained constant between cohorts (Q10, 27% vs. 27%, *p* = 0.58); however, institutional partnerships seem to mitigate discomfort more during the pandemic (Q15, 72% vs. 50%, *p* < 0.001). Figure 1 represents an inverted pyramid chart showing the progressive decline in the percentage of participants who reported comfort in sharing with recipients outside the circle of care. During the pandemic, it was evident that participants felt more comfortable sharing with all recipients except with for-profit commercial companies. More pandemic cohort patients preferred study-specific consent (Q5, 45% vs. 38%, *p* < 0.001), a larger percentage of pandemic cohort patients wished to track use of information and samples (Q18, 75% vs. 61%, *p* = 0.007), a greater percentage wanted to be notified with regard to study results (Q19, 83% vs. 70%, *p* = 0.012).

**Table 2:** Questionnaire responses by cohort

Question	Pandemic (N = 183)	Pre-pandemic (N = 222)	*p* value
Q1: Your health information is divided into several different sections (e.g. diagnoses test results and images such as x-rays or scans). Would you like to:			
Share all information	161 (88%)	177 (81%)	0.07
Share no information	4 (2%)	12 (5%)	
Share specific information	18 (10%)	30 (14%)	
Missing	0 (0.0%)	3 (1.4%)	
Q2: Your biological samples are classified into several different types (e.g. blood urine tissues). Would you like to:			
Share all information	163 (90%)	173 (79%)	**0.009**
Share no information	4 (2%)	18 (8%)	
Share specific information	15 (8%)	28 (13%)	
Missing	1 (0.5%)	3 (1.4%)	
Q3: There are many different areas of medical research (e.g. research on cancer diabetes reproductive disorders genetic disorders heart disease etc.). Would you like to:			
Share all information	157 (86%)	172 (79%)	0.17
Share no information	4 (2%)	9 (4%)	
Share specific information	21 (12%)	37 (17%)	
Missing	1 (0.5%)	4 (1.8%)	
Q4: When asked for consent to share your information or samples would you like to have an option to think about the decision and be asked again later?			
Yes	81 (44%)	107 (49%)	0.14
No	102 (56%)	111 (51%)	
Missing	0 (0.0%)	4 (1.8%)	
Q5: Your health information and samples are often requested for future studies. Would you like to:			
Broad consent	97 (53%)	119 (56%)	**< 0.001**
Study-specific consent	83 (45%)	80 (38%)	
Would not share	3 (2%)	14 (7%)	
Missing	0 (0.0%)	9 (4.1%)	
Q6: A CONTACT POOL may be created with patient names phone numbers and key pieces of health information. UHN Researchers with ethical approval for their studies could search this pool to find participants. Would you prefer to be:			
Asked for permission	105 (58%)	134 (63%)	0.06
Automatically entered	77 (42%)	80 (37%)	
Missing	1 (0.5%)	8 (3.6%)	
Q7: How comfortable are you with providing consent for your information or samples to be shared with Researchers within UHN?			
Comfortable/very comfortable	178 (97%)	183 (83%)	**< 0.001**
Neutral	5 (3%)	30 (14%)	
Uncomfortable/very uncomfortable	0 (0%)	8 (4%)	
Missing	0 (0.0%)	1 (0.5%)	
Q8: How comfortable are you with providing consent for your information or samples to be shared with Researchers at other hospital-based research institutes?			
Comfortable/very comfortable	155 (85%)	153 (70%)	**0.001**
Neutral	17 (9%)	44 (20%)	
Uncomfortable/very uncomfortable	11 (6%)	25 (11%)	
Q9: How comfortable are you with providing consent for your information or samples to be shared with Researchers at universities?			
Comfortable/very comfortable	152 (84%)	153 (70%)	**0.010**
Neutral	18 (10%)	39 (18%)	
Uncomfortable/very uncomfortable	11 (6%)	26 (12%)	
Missing	2 (1.1%)	4 (1.8%)	
Q10: How comfortable are you with providing consent for your information or samples to be shared with For-profit businesses (e.g. drug or insurance companies such as Pfizer)?			
Comfortable/very comfortable	50 (27%)	59 (27%)	0.58
Neutral	41 (22%)	49 (22%)	
Uncomfortable/very uncomfortable	92 (50%)	111 (51%)	
Missing	0 (0.0%)	3 (1.4%)	
Q11: How comfortable are you with providing consent for your information or samples to be shared with Not-for-profit businesses (e.g. Heart and Stroke Foundation of Canada)?			
Comfortable/very comfortable	124 (68%)	125 (57%)	0.09
Neutral	34 (19%)	50 (23%)	
Uncomfortable/very uncomfortable	24 (13%)	44 (20%)	
Missing	1 (0.5%)	3 (1.4%)	
Q12: How comfortable are you with providing consent for your information or samples to be shared Provincially (i.e. within Ontario)?			
Comfortable/very comfortable	127 (69%)	117 (53%)	**0.002**
Neutral	36 (20%)	58 (27%)	
Uncomfortable/very uncomfortable	20 (11%)	44 (20%)	
Missing	0 (0.0%)	3 (1.4%)	
Q13: How comfortable are you with providing consent for your information or samples to be shared Nationally (i.e. within Canada)?			
Comfortable/very comfortable	119 (65%)	116 (53%)	**0.022**
Neutral	40 (22%)	57 (26%)	
Uncomfortable/very uncomfortable	24 (13%)	46 (21%)	
Missing	0 (0.0%)	3 (1.4%)	
Q14: How comfortable are you with providing consent for your information or samples to be shared Internationally (i.e. around the world)?			
Comfortable/very comfortable	87 (48%)	85 (39%)	**0.024**
Neutral	50 (27%)	53 (24%)	
Uncomfortable/very uncomfortable	46 (25%)	81 (37%)	
Missing	0 (0.0%)	3 (1.4%)	
Q15: Sometimes for-profit companies develop partnerships with UHN and we work together on medical research projects. How comfortable are you consenting to share your information or samples for these projects			
Comfortable/very comfortable	131 (72%)	111 (50%)	**< 0.001**
Neutral	29 (16%)	48 (22%)	
Uncomfortable/very uncomfortable	23 (13%)	63 (28%)	
Q16: Sometimes for-profit companies ask UHN for health information or samples. How comfortable are you consenting to share your information or samples with these companies if UHN is not directly involved in their work?			
Comfortable/very comfortable	63 (35%)	68 (32%)	0.57
Neutral	36 (20%)	37 (17%)	
Uncomfortable/very uncomfortable	80 (45%)	110 (51%)	
Missing	4 (2.2%)	7 (3.2%)	
Q17: Sometimes medical research using health information or samples at UHN leads to discoveries that are commercialized and sold for-profit in the future. How do you feel about consenting to share your information or samples being involved in this?			
Comfortable/very comfortable	97 (53%)	81 (37%)	** < 0.001**
Neutral	48 (26%)	51 (23%)	
Uncomfortable/very uncomfortable	38 (21%)	88 (40%)	
Missing	0 (0.0%)	2 (0.9%)	
Q18: Would you like to be able to track who is using your information or samples and what they are using it for?			
Yes	137 (75%)	134 (61%)	**0.007**
No	43 (23%)	77 (35%)	
Not applicable. I would not share at all	3 (2%)	10 (4%)	
Missing	0 (0.0%)	1 (0.5%)	
Q19: Would you like to be notified with the results of studies that have used your information or samples?			
Yes	151 (83%)	55 (70%)	**0.012**
No	28 (15%)	57 (26%)	
Not applicable. I would not share at all	4 (2%)	10 (4%)	
Q20: If you do want to be notified of study results how would you like be notified?			
Online via an electronic patient portal	103 (56%)	76 (35%)	** < 0.001**
Online via email	44 (24%)	50 (23%)	
Standard mail	12 (7%)	43 (19%)	
I do NOT want to be notified of study results	19 (10%)	40 (18%)	
Not applicable. I would not share at all	5 (3%)	11 (5%)	
Missing	0	2	

The bold values are statistically significant

#### Multivariable regression models

Results from the multivariable regression models are reported in Tables 3 and 4. Table 3 includes results from the ordinal regression models used for questions assessing comfort level (Q7–Q17). Table 4 includes results from logistic regression models used for all other questions with responses that could be dichotomized (Q1 to Q6, Q18, Q19), along with the specific response for each question being modelled. After adjusting for demographics, patients in the pandemic cohort were still more likely than those in the pre-pandemic cohort to be comfortable with sharing all biological samples (Q2, OR = 2.11 [95% CI 1.03, 4.34], *p* = 0.042). They were also more comfortable sharing PHI with institutional researchers (Q7, OR = 8.35 [95% CI 2.85, 24.42], *p* < 0.001), and sharing PHI with researchers at other hospitals (Q8, OR = 2.50 [95% CI 1.49, 4.21], *p* < 0.001). Patients in the pandemic cohort were also more comfortable sharing provincially (Q12, OR = 2.01 [95% CI 1.32, 3.07], *p* = 0.001), nationally (Q13, OR = 1.66 [95% CI 1.10, 2.51], *p* = 0.0154), and with commercial/institutional partnerships (Q15, OR = 2.43 [95% CI 1.59, 3.72], *p* < 0.001). The proportional odds assumption held for all ordinal regression models. The pandemic cohort’s preference to track use of information and samples remained statistically significant (Q18, OR = 1.89 [95% CI 1.18, 3.03], *p* = 0.008), as did their preference to be notified with regard to study results (Q19, OR = 2.06 [95% CI 1.21, 3.51], *p* = 0.008).

**Table 3:** Results from multivariable ordinal regression models for Q7–Q17

Question	Covariate	Level	OR	95% CI	*p* value
Q7: How comfortable are you with providing consent for your information or samples to be shared with Researchers within UHN?	Cohort	Pandemic versus pre-pandemic	8.35	(2.85, 24.42)	0.0001
	Age group	50–74 versus 75+	1.33	(0.54, 3.24)	0.53
	Age group	< 49 versus 75+	0.36	(0.13, 1.03)	0.06
	Sex	Female versus male	0.82	(0.41, 1.67)	0.59
	Treatment phase	Follow-up versus treatment	1.36	(0.64, 2.88)	0.42
	Treatment phase	Pre-treatment versus treatment	0.49	(0.17, 1.42)	0.19
	Education	Post-sec. versus no/incomplete post-sec	1.98	(0.95, 4.13)	0.07
Q8: How comfortable are you with providing consent for your information or samples to be shared with Researchers at other hospital-based research institutes?	Cohort	Pandemic versus pre-pandemic	2.50	(1.49, 4.21)	0.0005
	Age group	50–74 versus 75+	0.87	(0.45, 1.68)	0.68
	Age group	< 49 versus 75+	0.54	(0.23, 1.24)	0.14
	Sex	Female versus male	0.87	(0.53, 1.41)	0.56
	Treatment phase	Follow-up versus treatment	1.55	(0.93, 2.59)	0.10
	Treatment phase	Pre-treatment versus treatment	0.64	(0.29, 1.46)	0.29
	Education	Post-sec. versus no/incomplete post-sec	1.14	(0.67, 1.92)	0.63
Q9: How comfortable are you with providing consent for your information or samples to be shared with Researchers at universities?	Cohort	Pandemic versus pre-pandemic	2.06	(1.23, 3.45)	0.006
	Age group	50–74 versus 75+	0.93	(0.49, 1.77)	0.83
	Age group	< 49 versus 75+	0.93	(0.40, 2.16)	0.86
	Sex	Female versus male	0.67	(0.41, 1.09)	0.11
	Treatment phase	Follow-up versus treatment	0.74	(0.44, 1.23)	0.24
	Treatment phase	Pre-treatment versus treatment	0.53	(0.23, 1.23)	0.14
	Education	Post-sec. Versus no/incomplete post-sec	1.36	(0.81, 2.30)	0.24
Q10: How comfortable are you with providing consent for your information or samples to be shared with For-profit businesses (e.g. drug or insurance companies such as Pfizer)?	Cohort	Pandemic versus pre-pandemic	1.01	(0.68, 1.50)	0.96
	Age group	50–74 versus 75+	0.68	(0.41, 1.14)	0.15
	Age group	< 49 versus 75+	0.99	(0.50, 1.97)	0.97
	Sex	Female versus male	0.73	(0.49, 1.07)	0.11
	Treatment phase	Follow-up versus treatment	0.62	(0.42, 0.93)	0.019
	Treatment phase	Pre-treatment versus treatment	0.36	(0.16, 0.79)	0.011
	Education	Post-sec. versus no/incomplete post-sec	0.82	(0.53, 1.25)	0.35
Q11: How comfortable are you with providing consent for your information or samples to be shared with Not-for-profit businesses (e.g. Heart and Stroke Foundation of Canada)?	Cohort	Pandemic versus pre-pandemic	1.67	(1.09, 2.55)	0.018
	Age group	50–74 versus 75+	0.51	(0.28, 0.93)	0.027
	Age group	< 49 versus 75+	0.44	(0.20, 0.94)	0.034
	Sex	Female versus male	0.75	(0.49, 1.13)	0.16
	Treatment phase	Follow-up versus treatment	0.89	(0.58, 1.37)	0.59
	Treatment phase	Pre-treatment versus treatment	0.45	(0.21, 0.94)	0.0342
	Education	Post-sec. versus no/incomplete post-sec	0.89	(0.56, 1.41)	0.61
Q12: How comfortable are you with providing consent for your information or samples to be shared Provincially (i.e. within Ontario)?	Cohort	Pandemic versus pre-pandemic	2.01	(1.32, 3.07)	0.0012
	Age group	50–74 versus 75+	0.98	(0.57, 1.69)	0.94
	Age group	< 49 versus 75+	0.71	(0.35, 1.45)	0.34
	Sex	Female versus male	0.82	(0.54, 1.23)	0.34
	Treatment phase	Follow-up versus treatment	0.88	(0.58, 1.35)	0.56
	Treatment phase	Pre-treatment versus treatment	0.69	(0.33, 1.47)	0.34
	Education	Post-sec. versus no/incomplete post-sec	0.84	(0.53, 1.32)	0.44
Q13: How comfortable are you with providing consent for your information or samples to be shared Nationally (i.e. within Canada)?	Cohort	Pandemic versus pre-pandemic	1.66	(1.10, 2.51)	0.0154
	Age group	50–74 versus 75+	1.06	(0.62, 1.81)	0.83
	Age group	< 49 versus 75+	0.87	(0.43, 1.76)	0.69
	Sex	Female versus male	0.75	(0.50, 1.12)	0.16
	Treatment phase	Follow-up versus treatment	0.97	(0.64, 1.47)	0.88
	Treatment phase	Pre-treatment versus treatment	0.55	(0.27, 1.15)	0.11
	Education	Post-sec. versus no/incomplete post-sec	0.84	(0.54, 1.31)	0.44
Q14: How comfortable are you with providing consent for your information or samples to be shared Internationally (i.e. around the world)?	Cohort	Pandemic versus pre-pandemic	1.47	(1.00, 2.16)	0.0494
	Age group	50–74 versus 75+	0.93	(0.56, 1.55)	0.78
	Age group	< 49 versus 75+	1.14	(0.58, 2.26)	0.71
	Sex	Female versus male	0.71	(0.48, 1.03)	0.074
	Treatment phase	Follow-up versus treatment	0.81	(0.54, 1.19)	0.28
	Treatment phase	Pre-treatment versus treatment	0.45	(0.22, 0.94)	0.03
	Education	Post-sec. versus no/incomplete post-sec	1.15	(0.76, 1.74)	0.52
Q15: Sometimes for-profit companies develop partnerships with UHN and we work together on medical research projects. How comfortable are you consenting to share your information or samples for these projects?	Cohort	Pandemic versus pre-pandemic	2.43	(1.59, 3.72)	< .0001
	Age group	50–74 versus 75+	0.94	(0.54, 1.61)	0.81
	Age group	< 49 versus 75+	0.77	(0.38, 1.57)	0.47
	Sex	Female versus male	0.96	(0.64, 1.44)	0.84
	Treatment phase	Follow-up versus treatment	0.74	(0.48, 1.13)	0.16
	Treatment phase	Pre-treatment versus treatment	0.73	(0.34, 1.56)	0.42
	Education	Post-sec. versus no/incomplete post-sec	1.15	(0.74, 1.79)	0.54
Q16: Sometimes for-profit companies ask UHN for health information or samples. How comfortable are you consenting to share your information or samples with these companies if UHN is not directly involved in their work?	Cohort	Pandemic versus pre-pandemic	1.16	(0.78, 1.71)	0.47
	Age group	50–74 versus 75+	0.90	(0.54, 1.52)	0.71
	Age group	< 49 versus 75+	0.72	(0.35, 1.47)	0.34
	Sex	Female versus male	0.61	(0.41, 0.90)	0.0121
	Treatment phase	Follow-up versus treatment	0.66	(0.44, 0.99)	0.0455
	Treatment phase	Pre-treatment versus treatment	0.43	(0.20, 0.93)	0.0318
	Education	Post-sec. versus no/incomplete post-sec	1.00	(0.65, 1.53)	0.99
Q17: Sometimes medical research using health information or samples at UHN leads to discoveries that are commercialized and sold for-profit in the future. How do you feel about consenting to share your information or samples being involved in this?	Cohort	Pandemic versus pre-pandemic	2.01	(1.36, 2.97)	0.0005
	Age group	50–74 versus 75+	1.21	(0.73, 2.03)	0.46
	Age group	< 49 versus 75+	0.92	(0.46, 1.82)	0.81
	Sex	Female versus male	0.52	(0.35, 0.76)	0.0009
	Treatment phase	Follow-up versus treatment	0.82	(0.55, 1.23)	0.34
	Treatment phase	Pre-treatment versus treatment	0.41	(0.19, 0.87)	0.0197
	Education	Post-sec. versus no/incomplete post-sec	1.36	(0.89, 2.07)	0.15

**Table 4:** Results from multivariable logistic regression models for Q1–Q6, Q18–Q19

Question	Outcome	Covariate	Level	OR	95% CI	*p* value
Q1: Your health information is divided into several different sections (e.g. diagnoses test results and images such as x-rays or scans). Would you like to:	1 = share all information; 0 = share some information	Cohort	Pandemic versus pre-pandemic	1.71	(0.87, 3.36)	0.12
		Age group	50–74 versus 75+	1.26	(0.51, 3.10)	0.61
		Age group	< 49 versus 75+	0.92	(0.30, 2.76)	0.88
		Sex	Female versus male	0.27	(0.13, 0.56)	0.0004
		Treatment phase	Follow-up versus treatment	1.25	(0.64, 2.43)	0.52
		Treatment phase	Pre-treatment versus treatment	2.1	(0.44, 9.97)	0.35
		Education	Post-sec. versus no/incomplete post-sec	0.84	(0.40, 1.79)	0.66
Q2: Your biological samples are classified into several different types (e.g. blood urine tissues). Would you like to:	1 = share all information; 0 = share some information	Cohort	Pandemic versus pre-pandemic	2.11	(1.03, 4.34)	0.042
		Age group	50–74 versus 75+	1.33	(0.51, 3.46)	0.56
		Age group	< 49 versus 75+	1.09	(0.34, 3.49)	0.89
		Sex	Female versus male	0.28	(0.13, 0.60)	0.0011
		Treatment phase	Follow-up versus treatment	1.27	(0.63, 2.54)	0.50
		Treatment phase	Pre-treatment versus treatment	3.97	(0.49, 32.12)	0.20
		Education	Post-sec. versus no/incomplete post-sec	0.55	(0.23, 1.30)	0.17
Q3: There are many different areas of medical research (e.g. research on cancer diabetes reproductive disorders genetic disorders heart disease etc.). Would you like to:	1 = share all information; 0 = share some information	Cohort	Pandemic versus pre-pandemic	1.84	(0.97, 3.48)	0.062
		Age group	50–74 versus 75+	0.69	(0.26, 1.81)	0.45
		Age group	< 49 versus 75+	0.57	(0.18, 1.77)	0.33
		Sex	Female versus male	0.22	(0.11, 0.44)	< .0001
		Treatment phase	Follow-up versus treatment	0.98	(0.53, 1.83)	0.95
		Treatment phase	Pre-treatment versus treatment	2.07	(0.44, 9.79)	0.36
		Education	Post-sec. versus no/incomplete post-sec	0.7	(0.33, 1.46)	0.34
Q4: When asked for consent to share your information or samples would you like to have an option to think about the decision and be asked again later?	1 = option to re-consider; 0 = no option to re-consider	Cohort	Pandemic versus pre-pandemic	0.85	(0.56, 1.28)	0.43
		Age group	50–74 versus 75+	1.64	(0.94, 2.87)	0.083
		Age group	< 49 versus 75+	2.28	(1.08, 4.81)	0.0307
		Sex	Female versus male	1.05	(0.69, 1.58)	0.83
		Treatment phase	Follow-up versus treatment	1.16	(0.76, 1.78)	0.48
		Treatment phase	Pre-treatment versus treatment	1.32	(0.60, 2.92)	0.49
		Education	Post-Sec. versus no/incomplete post-sec	0.87	(0.55, 1.36)	0.53
Q5: Your health information and samples are often requested for future studies. Would you like to:	1 = broad consent; 0 = study-specific consent	Cohort	Pandemic versus pre-pandemic	0.78	(0.50, 1.21)	0.27
		Age group	50–74 versus 75+	0.61	(0.34, 1.12)	0.11
		Age group	< 49 versus 75+	0.43	(0.20, 0.95)	0.0366
		Sex	Female versus male	0.55	(0.36, 0.85)	0.0068
		Treatment phase	Follow-up versus treatment	0.84	(0.54, 1.31)	0.45
		Treatment phase	Pre-treatment versus treatment	1.19	(0.49, 2.90)	0.70
		Education	Post-sec. versus no/incomplete post-sec	1.18	(0.73, 1.90)	0.51
Q6: A contact pool may be created with patient names phone numbers and key pieces of health information. UHN Researchers with ethical approval for their studies could search this pool to find participants. Would you prefer to be:	1 = automatically entered into a contact pool; 0 = asked for permission	Cohort	Pandemic versus pre-pandemic	1.12	(0.73, 1.72)	0.60
		Age group	50–74 versus 75+	0.75	(0.42, 1.32)	0.32
		Age group	< 49 versus 75+	0.59	(0.27, 1.27)	0.18
		Sex	Female versus male	0.74	(0.49, 1.14)	0.17
		Treatment phase	Follow-up versus treatment	0.84	(0.55, 1.31)	0.45
		Treatment phase	Pre-treatment versus treatment	0.97	(0.42, 2.26)	0.95
		Education	Post-sec. versus no/incomplete post-sec	1.76	(1.09, 2.85)	0.0204
Q18: Would you like to be able to track who is using your information or samples and what they are using it for?	1 = wanting to track information/sample use; 0 = not wanting to track information/sample use	Cohort	Pandemic versus pre-pandemic	1.89	(1.18, 3.03)	0.0079
		Age group	50–74 versus 75+	1.98	(1.12, 3.51)	0.0185
		Age group	< 49 versus 75+	2.68	(1.15, 6.26)	0.0223
		Sex	Female versus male	1.12	(0.71, 1.78)	0.61
		Treatment phase	Follow-up versus treatment	1.61	(1.00, 2.61)	0.0498
		Treatment phase	Pre-treatment versus treatment	1.3	(0.54, 3.13)	0.55
		Education	Post-sec. versus no/incomplete post-sec	1.18	(0.72, 1.94)	0.50
Q19: Would you like to be notified with the results of studies that have used your information or samples?	1 = wanting to be notified of study results; 0 = not wanting to be notified of study results	Cohort	Pandemic versus pre-pandemic	2.06	(1.21, 3.51)	0.0082
		Age group	50–74 versus 75+	1.56	(0.84, 2.88)	0.16
		Age group	< 49 versus 75+	2.67	(0.99, 7.22)	0.053
		Sex	Female versus male	1.16	(0.70, 1.94)	0.56
		Treatment phase	Follow-up versus treatment	1.59	(0.93, 2.72)	0.092
		Treatment phase	Pre-treatment versus treatment	0.84	(0.34, 2.08)	0.70
		Education	Post-sec. versus no/incomplete post-sec	1.02	(0.59, 1.76)	0.95

The outcome column outlines how the outcome was coded for each question

### Focus groups

#### Theme 1 mixed preferences and a diverse array of reasons justifying them emerged with respect to who should make PHI-sharing decisions, opt-in versus opt-out approaches for a research contact pool, and broad versus specific consent strategies

Some participants noted that hospital committees would be necessary to render informed and ethically defensible decisions because *expert knowledge is needed*, and this will *expedite research*.

I agree with the committee [making decisions]… there are the people involved in the law and ethics who come in that their judgment is much better than mine. FG1, P2 (Focus Group 1, Patient 2)

As a patient, I want to advance the research, as quickly as we can. I don't want that to become a deterrent FG1, P3

However, individual consent was felt to be important to *respect individual preferences*, and there remained a notable *lack of trust* surrounding privacy and uses of information.

The nature of some studies that could be done you might not be in agreement with. There are people who have very strong views on certain types of research. FG2, P1

Why should I believe you that my information is not going to be used in ways that I don't know about? Why should I believe you that my name won’t go up there or my personal information won’t be attached to the samples or research? FG2, P3

So some of this information can go to [how to] cure cancer, and the other could go to, how to create maybe a biological weapon that would create cancer. FG1, P2

In a similar sense, opt-out approaches to assemble a contact pool were acknowledged by participants to be important/valuable for researchers to access the *largest participant group*, because some participants maintained that the *communal benefit of research* should outweigh individual privacy risks.

You might get a lot of no’s, and eliminate the number of people that the researchers can select from, and they might need that. FG2, P3

I do think the benefits of a larger contact pool would far outweigh personal freedom in this matter. FG2, P3

However, due to both legal and ethical/pragmatic concerns, opt-in (i.e. being asked for permission before research contact pool entry) was often still preferred, as opt-out (i.e., automatic entry with a chance to later be removed) was perceived to be a *breach of privacy* and was associated with a *loss of control*.

I think that if we are all automatically entered, there might be a breach of privacy…not all patients are updating even their family that they are sick… I'm not sure legally you can do that.

You've already lost control of your health and you're now at the mercy of whatever has to be done next. To lose control over what's happening around you and the information that's being collected… there was just enough on my shoulders. FG1, P2

Finally, the benefits of broad consent were recognized for *practical reasons* with many acknowledging logistical struggles with providing information on a study-specific basis.

In an ideal world, it would be nice to be asked. But, again, we go back to the question of practicality. FG2, P1

It would be a nightmare for researchers to call everybody, every time they want to submit their information. FG2, P3

However, many participants still expressed preferences for study-specific consent due to *ethical concerns* about potential research uses, and so they indicated a preference to be *informed*, *educated*, and *given a choice*.

I may have ethical concerns about the research being done, or who's doing the research…there are certain organizations that I don't ever want to have access to my information like insurance companies…I may consider the research being done unethical. FG1, P1

I’d need more safeguards than asking one person once and then coming across their tissues 110 years later. FG3, P1

Knowing the study would inform me, give me a choice, and also educate me about how our information is being used. FG1, P3

#### Theme 2 strong hesitancy and concerns were expressed around commercial uses of PHI

With regards to commercial entities, participants often expressed *not wanting to share* their PHI for this purpose at all, or to be given *detailed information* so they could make decisions on a study-specific basis.

I would never ever want any information going to a company. FG1, P1.

I want to know each and every time they need my information...And I would probably want to know way more information. I would want to know who's the sponsor? Who pays? Who is doing the studies? Where does it go? FG1, P2

Others stressed their view that *de-identification did not lessen discomfort*.

Data, whether it has your name or not, can be used against you…I mean, do you have a pre-existing condition? …Oh, I'm sorry, we won't insure you. So that data, even nameless, unknown, is already being used against us. FG1, P3

Reasons behind participant discomfort often centered on companies, which respondents suggested may not be held to the same *legal and ethical norms* as academic institutions or hospitals, with profits being their primary interest.

The problem with companies is that they're not obliged to the same legal and ethical laws and norms as academic and non-profit organizations like academic centers, institutions and hospitals. So once my information goes there, it's already like I’m a little bit giving it out the door…I want to have way more control over that…There might be benefit of them curing cancer, but it's not their original intention. Their original intention is money. FG1, P3

By and large we’d like to think [companies] all do things ethically with humanity in mind but quite frankly that is not the nature of the world. There’s a lot of industries that do not have humanity in mind, they actually have profit and shareholders in mind. FG3, P3

My only concern would be [some] companies. I might not agree with or I might not want my information [shared] with them. I would not want them to benefit from my information, and by that I mean, companies that have any racist policies or something like that, that I don't agree with or I don't see as ethical. I would not want to help them with my information. FG2, P3

Many also noted significant concerns around *inequity* related to commercial uses of PHI.

I’ve seen situations where patients were deprived of treatment as a result of the inability to pay…So there’s a problem with a company putting something out like that, that has actually benefited from public support in terms of samples. FG4, P3

I can’t allow companies using my information for them to make money and then the people that I want to help don’t get access to that. FG4, P2

Some participants did report supporting commercial uses of PHI because the work has the potential to benefit patients and blocking it could slow progress of important research.

I have no problem with [them] getting money from that. Because again, I know that this will help people. This will improve treatment. We live in a world where everything runs on money. So, I would support that. FG2, P3

Yeah, I’m okay with my information going to companies for profit cause yeah, they’re major producers of most of the drugs and so I’m all for any type of research. FG4, P1

So the price we pay for holding companies up I think, is to put a block on research that benefits us. FG4, P3

#### Theme 3 transparency through the provision of information and multiple consent options was identified as essential to building trust

The ability to *be informed* and to *receive study results* was strongly preferred by nearly all participants.

I think trust comes from asking the patient's permission. And perhaps even if they say, yes, that they agree that at some point, information comes back to say, what the results are? Because we go into a study, but we don't hear that about, like, what happened? Did it fail? Were you successful?…In politics, we keep hearing ‘be transparent’. So, in the medical profession, be transparent. Especially with for-profit companies. If they want to build trust, we need to see evidence over time…it would help to see how they used it and you know, how we may benefit as patients in the long run… FG1, P3

Information is the key I think. The more information there is, the more trust people have in their health care provider. FG2, P3

Getting permission from each individual patient, per the type of information they're prepared to share. Is there more information on the study? If it meets our ethical concerns? Can we trust who it's going to and for what purposes?…If it's a more open sharing of information with the patients, then there may be more trust developed. FG1, P1

And also [we need] information on the nature of the studies. People are curious. They'd like to know where it is and what it's being used for. FG2, P1

The more informed we are, the more likely we are to help. FG2, P2

About two and a half years ago, I was in palliative care….At that point, if I had been in a study, and they had results for me, it would have been encouraging. I think to know that something positive is coming out of collecting your information…To know that you've somehow made a difference. FG1, P3

Many participants suggested, and supported, offering *multiple consent options upon entry* into the healthcare system, and to have the *opportunity to alter preferences* throughout their care journey.

What I would suggest is that anytime somebody comes into the hospital for any form of treatment is that everybody's filling out forms in the first place that says, ‘yes, I'm happy to have my information shared.’ FG2, P2

It would be nice to develop an implementation pathway that any individual patient coming into the [hospital]…they are asked and signed and notified that their information in the affirmative could be used for research, like right up front. FG3, P3

I think having a pop-up box, like maybe annually…So, in the beginning again, I might have said no. And then along with my treatment becoming more appreciative, how it's helping you because I'm learning more about [the hospital]…building that trust with your oncologist and your oncology team, it changes your outlook. So at this point, I might want to change. I might want to help. FG2, P3

I feel strongly about having the chance to say no, at the start. FG2, P2

[Should be asked] right at the beginning when you register…So you might say yes at the beginning and then along the way you might change your mind or there might be something that you don't want shared. FG2, P3

If it's all on your personal portal with the options, it makes life so easy, and you know people can change it as they go and actually that makes it less bureaucratic because, you know, it's all there digitally. FG2, P2

## Discussion

This study’s findings show that cancer patients at a large Canadian hospital were substantially more comfortable sharing PHI during a pandemic compared to pre-pandemic times. This overall finding reflects an altruistic desire of some patients to contribute to the advancement of medical science during a pandemic and supports the notion that provisions to modify/revisit consent policies during times of crisis may be justified. Our findings however, also underscore the continuing need for organizations to exercise caution around the continual concerns over sharing data for commercial uses even during a pandemic. Commercial entities were the only potential recipients of PHI with whom participants did not feel comfortable (~ 50% discomfort levels before and during the pandemic). Previous research has documented that patients are reluctant to share PHI for commercial purposes [10–12, 17, 18]. Our focus group findings demonstrate that this heightened discomfort is owing to the perception that commercial companies are driven mainly by profit, and less by humanitarian motives (i.e., as opposed to academic hospitals). Our focus group participants opined that companies are not held to the same legal and ethical standards as health care institutions, and substantial concerns were raised regarding inequity in access to care or research-related opportunities or options. It was noted that while PHI is obtained from *all* patients to develop novel therapies, not everyone however, could afford them when needed. While some of these issues may seem insurmountable, innovative solutions emerged around how trust needs to be built, and this trust can serve as a framework for healthcare organizations to adapt their consent policies and processes. Participants underscored that transparency was key to building trust, in that they desired more information, wanted to be asked for permission, and indicated a desire to be able to change preferences over time. Digital dynamic consent platforms (e.g., [19–21]) are clearly essential to meeting the needs of patient-centred research-related decision-making, and should be adopted on a broad scale.

Our results regarding mixed preferences around individual versus committee consent decisions, opt-in versus opt-out approaches, and broad versus specific consent strategies echo similar reports that preferences vary with the circumstances of the purpose, user, and the controls placed on use throughout the literature (e.g., [13, 22]); further underscoring the complex and heterogeneous nature of these issues. It is evident that clarity and transparency are of paramount importance to patients, as is the provision of information upon entry into the healthcare system, the option to choose whether to share PHI outside the circle of care, and the ability to track and change preferences over time. Attention to these considerations will help institutions in devising consent policies that will meet the complex and diverse needs of our patients, and move towards the implementation of consent processes that are dynamic, and reflecting individual patient values and preferences. Indeed, governments around the world are revising laws around data usage; the EU’s GDPR [2] is recognized as amongst the most stringent privacy and security law in the world.

We acknowledge that this study was not without limitations, most notably the 6% response rate. While we acknowledge that this is a low rate, this was not unexpected during a time of global crisis; many people were adjusting to significant challenges related to employment, childcare, and lockdown restrictions. Despite this, we were still able to reach theme saturation (i.e. no new themes emerged from the data as verified by three independent coders; Malterud, 2015) [23], and indeed, it has been reported that 3–6 focus groups are sufficient to reach saturation in similar qualitative studies in the health care field [24]. It is also important to note that the participants were a specific sample (i.e., cancer patients receiving care from a research-intensive hospital who have consented to being contacted for research purposes), and may not be representative of a broader sample of patients with different diagnoses or international locations. These perceptions were also gleaned during Wave 1 of the pandemic, and it is possible that preferences may shift over the course of multiple pandemic waves. These areas represent important avenues for future inquiry.

In conclusion, the Canadian Institute for Health Research (CIHR) Tri-Council Policy Statement for Ethical Conduct of Research with Humans [1] articulates three pillars of consent: informed, voluntary, and ongoing; this is precisely what our participants desired regarding the use of the PHI outside the circle of care. Organizations have the responsibility to meet these needs with respect to sharing PHI by adopting digital dynamic consent platforms. If this were implemented on a broad scale, patients’ discomfort in sharing PHI with commercial entities (particularly when their healthcare institution is not involved in the work) might be assuaged. The COVID-19 pandemic has been an unpreceded time, revealing the power of medical science to save lives and to advance the common good. At the same time, it has highlighted profound social inequities and the high costs associated with mistrust in healthcare experts and researchers. Opportunities to demonstrate respect for patients’ values and preferences (e.g. dynamic consent approaches) must be undertaken if our communities are to form teams united by a shared vision to advance science and research for the benefit of our patients and society.

## Supplementary Information

**Additional file 1**. Patient Consent Preferences Survey.

**Additional file 2**. Patient Consent Preferences Focus Group Guide.

## Data Availability

The datasets generated and/or analyzed during the current study are not publicly available due to the potentially identifying nature of transcripts if viewed in full. They are available from the corresponding author on reasonable request.

## Abbreviations

PHI: Personal health information
CRC: Clinical research coordinator
REB: Research Ethics Board
UHN: University Health Network
