# Supplementary material for: Patient consent preferences on sharing personal health information during the COVID-19 pandemic: “the more informed we are, the more likely we are to help”
Source: BMC Med Ethics. 2022 May 20;23:53. doi: 10.1186/s12910-022-00790-z (PMC9122733; doi:10.1186/s12910-022-00790-z)
Supplement: Supplementary file 1 — Additional file 1. Patient Consent Preferences Survey. [file 12910_2022_790_MOESM1_ESM.docx]

**Appendix 1. Survey**

Hello and welcome,

You are being invited to participate in this survey because you are a patient at the Princess Margaret Cancer Centre within the University Health Network (UHN). It will only take about 10 minutes and your answers are anonymous (i.e., not linked to your name or medical record).

During your care at UHN, health information (e.g., your medical record with diagnoses, test results, and images such as x-rays), and biological samples (e.g., blood, tissues, or bodily fluids drawn for medical procedures) are collected and stored. The main purpose of collecting and storing your information and samples is for your own treatment and care.

Your health information and biological samples are also very valuable for other purposes. They can be used to help find new ways to detect, treat, prevent, and possibly cure many health-related problems such as cancer, diabetes, and heart disease. Researchers within UHN, and at other hospital-based research institutes, universities, and health-related companies request access to your information and samples for these reasons. The purpose of this survey is to help us understand your preferences on the way we ask for your consent to share.

When you consent to sharing your health information or samples, security measures are in place to protect your privacy including removing your name and anything that could directly identify you. There is still a small risk that donated information or samples could be traced back to you.

The choice to do this survey is completely up to you. Your decision will not affect your treatment or care in any way. During the completion of the survey, you are free to withdraw at any time and your answers will be deleted. There are no known risks or direct benefits to participating. If you consent to participating in this survey, please answer the questions on the following pages.

Thank you very much!

**Section 1: Asking for your Consent to Share**

1. Your health information is divided into several different sections (e.g., diagnoses, test results, and images such as x-rays or scans). Would you like to:
   1. Share ALL of your health information for research
   2. Share NONE of your health information for research
   3. CHOOSE SPECIFIC PARTS of your health information to share for research
2. Your biological samples are classified into several different types (e.g., blood, urine, tissues). Would you like to:
   1. Share ALL your biological samples for research
   2. Share NONE of your biological samples for research
   3. CHOOSE SPECIFIC PARTS of your biological samples to share for research
3. There are many different areas of medical research (e.g., research on cancer, diabetes, reproductive disorders, genetic disorders, heart disease, etc.). Would you like to:
   1. Share with ALL areas of research
   2. Share with NO areas of research
   3. CHOOSE SPECIFIC AREAS of research to share with
4. When asked for consent to share your information or samples, would you like to have an option to think about the decision and be asked again later?
   1. Yes
   2. No
5. Your health information and samples are often requested for future studies. Would you like to:
   1. Be asked for consent ONCE for ALL FUTURE STUDIES. You are not given study details
   2. Be contacted with details about each study and asked for consent EACH TIME
   3. Not Applicable. I would NOT share at all
6. A CONTACT POOL may be created with patient names, phone numbers, and key pieces of health information (e.g., “breast cancer” or “depression” or “family support clinic”). UHN Researchers with ethical approval for their studies could search this pool to find participants. Would you prefer to be:
7. ASKED FOR YOUR PERMISSION and only entered in the pool if you say yes
8. AUTOMATICALLY ENTERED in the pool. You are given a form with a number to call if you’d like to be removed

**Section 2: Recipients**

With security measures in place to protect your privacy including removing your name and anything that could directly identify you (though there is still a small risk that your information or samples could be traced back to you), please tell us how comfortable you feel sharing your information or samples with the following recipients.

How comfortable are you with providing consent for your information or samples to be shared with **Researchers within UHN?**

- 1. Very Comfortable
  2. Comfortable
  3. Neutral
  4. Uncomfortable
  5. Very Uncomfortable

How comfortable are you with providing consent for your information or samples to be shared with **Researchers at other hospital-based research institutes**?

1. Very Comfortable
2. Comfortable
3. Neutral
4. Uncomfortable
5. Very Uncomfortable

How comfortable are you with providing consent for your information or samples to be shared with **Researchers at universities**?

1. Very Comfortable
2. Comfortable
3. Neutral
4. Uncomfortable
5. Very Uncomfortable

How comfortable are you with providing consent for your information or samples to be shared with **For-profit businesses (e.g., drug or insurance companies such as Pfizer)**?

1. Very Comfortable
2. Comfortable
3. Neutral
4. Uncomfortable
5. Very Uncomfortable

How comfortable are you with providing consent for your information or samples to be shared with **Not-for-profit businesses (e.g., Heart and Stroke Foundation of Canada)**?

1. Very Comfortable
2. Comfortable
3. Neutral
4. Uncomfortable
5. Very Uncomfortable

How comfortable are you with providing consent for your information or samples to be shared **Provincially (i.e., within Ontario)?**

1. Very Comfortable
2. Comfortable
3. Neutral
4. Uncomfortable
5. Very Uncomfortable

How comfortable are you with providing consent for your information or samples to be shared **Nationally (i.e., within Canada)?**

1. Very Comfortable
2. Comfortable
3. Neutral
4. Uncomfortable
5. Very Uncomfortable

How comfortable are you with providing consent for your information or samples to be shared **Internationally (i.e., around the world)?**

1. Very Comfortable
2. Comfortable
3. Neutral
4. Uncomfortable
5. Very Uncomfortable

**Section 3: Commercialization**

When medical discoveries are made such as new treatments or cures for diseases, commercialization (i.e., selling for money) helps the discoveries to reach and help a great number of people. It also means that researchers and commercial companies may benefit financially. Patients who contribute their health information or samples do not get any money from this commercialization. We would like to understand how you feel about consenting to your health information or samples being used for projects that involve commercialization.

1. Sometimes for-profit companies (e.g., drug or insurance companies) develop partnerships with UHN and we work together on medical research projects. How comfortable are you consenting to share your information or samples (with your name and direct identifiers removed) for these projects?
2. Very Comfortable
3. Comfortable
4. Neutral
5. Uncomfortable
6. Very Uncomfortable
7. Sometimes for-profit companies (e.g., drug or insurance companies) ask UHN for health information or samples. How comfortable are you consenting to share your information or samples (with your name and direct identifiers removed) with these companies if UHN is not directly involved in their work?
8. Very Comfortable
9. Comfortable
10. Neutral
11. Uncomfortable
12. Very Uncomfortable
13. Sometimes medical research using health information or samples at UHN leads to discoveries that are commercialized and sold for-profit in the future. How do you feel about consenting to share your information or samples (with your name and direct identifiers removed) being involved in this?
14. Very Comfortable
15. Comfortable
16. Neutral
17. Uncomfortable
18. Very Uncomfortable

**Section 4: Tracking Sharing & Results**

1. Would you like to be able to track who is using your information or samples and what they are using it for?
   1. Yes
   2. No
   3. Not Applicable. I would not share at all
2. Would you like to be notified with the results of studies that have used your information or samples?
   1. Yes
   2. No
   3. Not Applicable. I would not share at all
3. If you do want to be notified of study results, **how** would you like to be notified?
   1. Online via an electronic patient portal (i.e., a secure website that allows you to track your personal health record)
   2. Online via email
   3. Standard mail
   4. I do NOT want to be notified of study results
   5. Not Applicable. I would not share at all

**Section 5: Background Questions**

Please answer the following questions on your background. Thank you very much for your participation!

1. Which clinic are you receiving treatment in?
2. Breast
3. Prostate
4. Lung
5. Thyroid
6. Other
7. What stage of your journey are you currently at?
   1. Pre-treatment
   2. Treatment
   3. Follow-up
8. Age
9. 18-34
10. 35-49
11. 50-74
12. 75+
13. Rather not say
14. Sex
15. Male
16. Female
17. Other
18. Rather not say
19. Ethnicity
20. Canadian
21. European Canadian
22. African Canadian
23. Asian Canadian
24. First Nations
25. Other
26. Rather not say
27. Highest level of education
28. High school or less
29. Some post-secondary training (i.e., trade, college, university)
30. Completed trade/college diploma
31. Completed university degree
32. Completed post-graduate university degree (e.g., MA, MSc, PhD)
33. Rather not say
34. Household income ($)
35. <40,000/year
36. 40,000-60,000/year
37. 60,000-80,000/year
38. 80,000-100,000/year
39. >100,000+/year
40. Rather not say
