# Supplementary material for: Patient consent preferences on sharing personal health information during the COVID-19 pandemic: “the more informed we are, the more likely we are to help”
Source: BMC Med Ethics. 2022 May 20;23:53. doi: 10.1186/s12910-022-00790-z (PMC9122733; doi:10.1186/s12910-022-00790-z)
Supplement: Supplementary file 2 — Additional file 2. Patient Consent Preferences Focus Group Guide. [file 12910_2022_790_MOESM2_ESM.pdf]

Appendix 2:
The Use of Your Health Information Outside Your Own Care

How do you feel?

UHN
Toronto General
Toronto Western
Princess Margaret
Toronto Rehab
Michener Institute

Your Health Information (HI)

- During your care at UHN, information about you including **data** (e.g. medical record), **images** (e.g. x-rays) and **biological samples** (e.g. blood) are collected and stored

  - **Main purpose = for your own care!**

UHN Toronto General Toronto Western Princess Margaret Toronto Rehab Michener Institute

Your Health Information (HI)

- HI is also very valuable for **other purposes**:
  - To detect, treat, prevent, or possibly cure diseases

- Researchers at UHN and other hospitals, universities, and companies **request access** to your de-identified (name removed) HI for these purposes

UHN
Toronto General
Toronto Western
Princess Margaret
Toronto Rehab
Michener Institute

Protections

- When HI is shared, **security measures** are in place to protect your privacy including removing your name and anything that could directly identify you

- Laws, ethical guidelines, and hospital committees are in place to govern the sharing of HI
  - Personal Health Information Protection Act (PHIPA)
  - Tri-Council Policy Statement (TCPS2) – Ethical Conduct for Research Involving Humans
  - Hospital Research Ethics Boards (REBs)

Toronto General
Toronto Western
Princess Margaret
Toronto Rehab
Michener Institute

Your Thoughts & Needs…

- UHN's primary value: **The needs of patients come first!**
  - How do you feel about the use of your HI outside your own care?
  - This information will help us make sure our policies for sharing HI meet the needs of our patients

UHN
Toronto General
Toronto Western
Princess Margaret
Toronto Rehab
Michener Institute

A Complicated Issue!

- Some **concerns** have been expressed about sharing HI
  - Control – who decides if and how HI is shared?
  - Consent – are patients given what they need to make informed choices?
  - Privacy – risk of others finding out your private health information
  - Commercialization – companies making money from HI

- It also has many **benefits** for our society
  - Lots of HI allows us to do lots of research!
  - Research leads to medical discoveries that improve and save lives
  - Treatments and even cures for many diseases can be found

UHN Toronto General Toronto Western Princess Margaret Toronto Rehab Michener Institute

Questions so far?

- Feel free to ask any time!

UHN
Toronto General
Toronto Western
Princess Margaret
Toronto Rehab
Michener Institute

4 Discussion Topics for Today

1. **Who should decide** if HI is shared?
2. How should we **contact** patients to participate in research?
3. What information do you need about **future studies** that may use your HI?
4. How do you feel about **companies** using your HI?

Toronto General
Toronto Western
Princess Margaret
Toronto Rehab
Michener Institute

1. Sharing PHI – who decides?

The process right now:

- Hospital committees (e.g., Research Ethics Boards; REBs) make decisions on who can access your HI. These committees are made up of specialists in medicine, law, and ethics.

- Current health privacy laws allow these committees to permit the sharing of HI without asking each patient for permission if it has been de-identified (no name) and the need to use it is justified.

What are your thoughts?

- How do you feel about hospital committees making these decisions on patients' behalf?

- If it were possible, do you think each patient should be informed and asked for permission first to use their HI outside of their care, even if their name has been removed?

Some things to consider…

- **If hospitals make the decisions:**
  - Benefits: Lots of data is available for research. More data = more research = more benefit for society.
  - Concerns: Patients are not informed or given the choice to say no. Some patients may not agree with certain research or have privacy concerns.

- **If each patient is asked for permission:**
  - Benefits: Patients are informed. The individual choices of each patient are respected.
  - Concerns: When patients say no, that data will not be available for research. Less data = less research = less benefit for society.

UHN Toronto General Toronto Western Princess Margaret Toronto Rehab Michener Institute

2. How should we contact patients to participate?

- A **CONTACT POOL** may be created with patient names, phone numbers, and key pieces of health information (e.g., “breast cancer” or “depression” or “family support clinic”). UHN Researchers with ethical approval for their studies could search this pool to find participants.

- Would you prefer to be **asked for permission** before being entered OR be **automatically entered** with the chance to opt-out? **Why?**

UHN Toronto General Toronto Western Princess Margaret Toronto Rehab Michener Institute

Some things to consider…

- **Asked for permission**: Patients are informed and have more control, but it’s harder for researchers to find participants (takes time and resources)

- **Automatically entered**: Easier for researchers to find participants, but patient name & diagnosis is shared outside the circle of care without direct consent

3. Future Studies

- Once researchers have your HI, it can be used for many different studies in the future that have not been designed yet.

- Would you like to be asked **one time** to use your HI for all future research OR be re-contacted with study details and asked for permission **every time**?

- If you wanted to be contacted every time, you would likely receive a notification online (e.g., through the UHN patient portal) with a brief study description, and you could click yes/no to the use of your de-identified HI for the study.

UHN Toronto General Toronto Western Princess Margaret Toronto Rehab Michener Institute

Some things to consider

- **Asked one time for all future research:** Easier for researchers to conduct their work (more research can be done) but patients have less information and control

- **Asked for consent for every study:** Patients have more information and control but it's harder for researchers to conduct their work (less research can be done)

UHN Toronto General Toronto Western Princess Margaret Toronto Rehab Michener Institute

4. Sharing HI with companies: How do you feel?

- **HI (without name) is often requested by commercial companies**
- How do you feel about UHN sharing your HI (without name) with companies?

Toronto General
Toronto Western
Princess Margaret
Toronto Rehab
Michener Institute

Some things to consider…

- **Benefits** to sharing HI with companies:
  - Companies use HI to develop new drugs and technologies that can help treat and even cure many major health problems such as cancer
  - This work can save and improve many lives

- **Concerns** with sharing HI with companies:
  - Companies make money using HI but patients are not paid to share it
  - HI could be linked with other data to re-identify patients (rare but possible)
  - Patients may not be aware that their HI is being shared with companies
  - Patients may not be given the choice whether or not to share with companies

Sharing PHI with companies

- Does it make a difference if revenue is generated for UHN?
- Does UHN involvement in the work make a difference?
- Does type of company make a difference? (insurance *vs.* drug)
- Does type of HI make a difference? (medical records with diagnoses *vs.* x-rays *vs.* blood samples)

Building Trust – How?

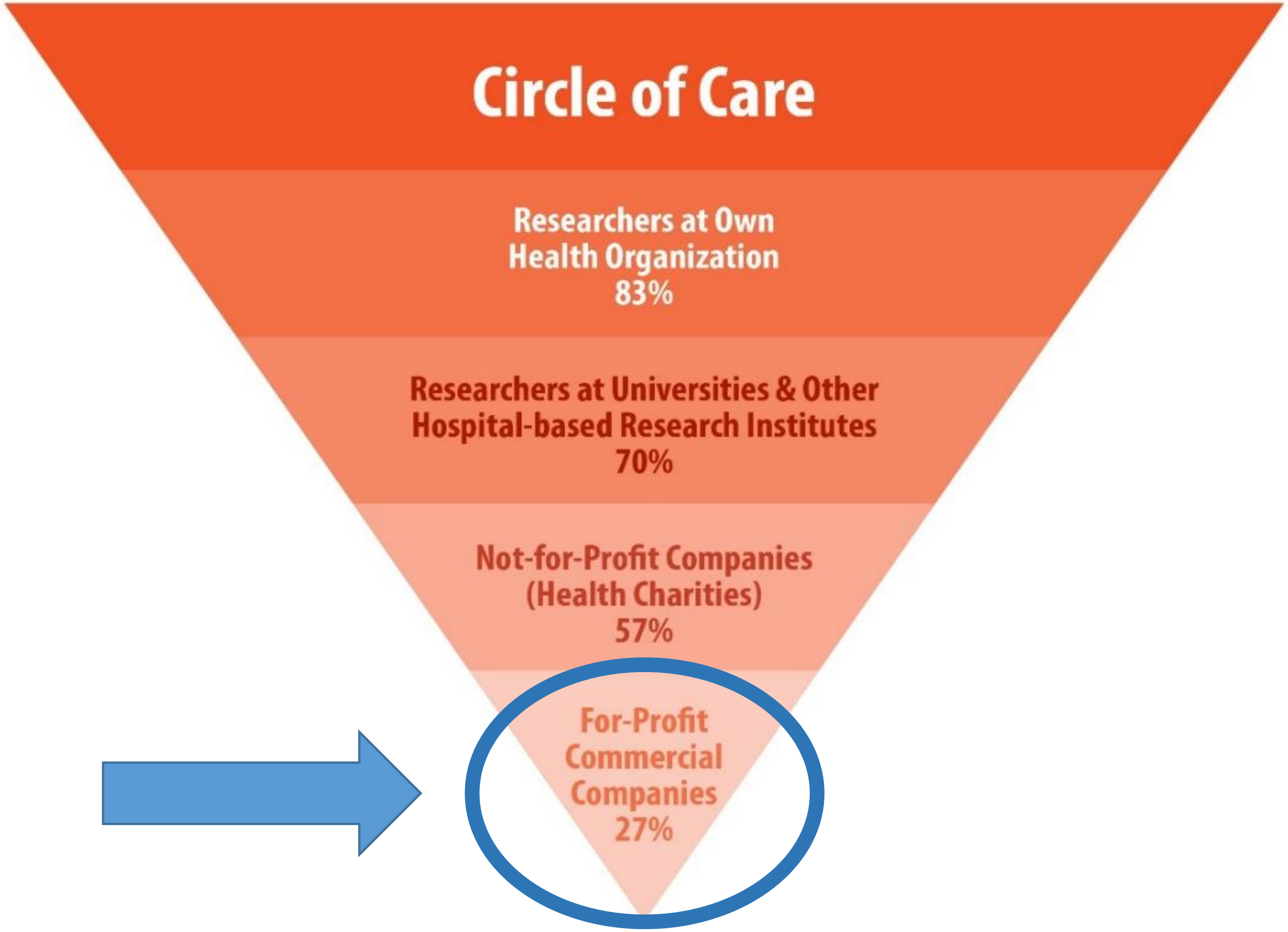

- **In a previous study - only 27% of patients reported comfort sharing HI with for-profit companies**
- **What could lessen that discomfort?**
- **Can trust be built? How?**
  - Asking patients for permission first?
  - Providing more information?
  - Anything else?

Toronto General
Toronto Western
Princess Margaret
Toronto Rehab
Michener Institute

THANK YOU!

Your participation is greatly appreciated!

UHN Toronto General Toronto Western Princess Margaret Toronto Rehab Michener Institute
